# PREVALENCE OF JOINT PAIN AND OSTEOARTHRITIS IN OBESE BRAZILIAN POPULATION

**DOI:** 10.1590/0102-672020180001e1344

**Published:** 2018-03-01

**Authors:** Daniel Moreira PACCA, Gustavo Constantino DE-CAMPOS, Alessandro Rozin ZORZI, Elinton Adami CHAIM, Jõao Batista DE-MIRANDA

**Affiliations:** 1 Orthopedics Department; 2 Surgery Department, State University of Campinas, Campinas, SP, Brazil

**Keywords:** Osteoarthritis, Obesity, Bariatric surgery, Osteoartrite, Obesidade, Cirurgia bariátrica

## Abstract

**Background:**

High body mass index, as well as maintaining this condition for a long period of time, are important risk factors for the development of osteoarthritis.

**Aim:**

To determine joint pain and osteoarthritis prevalence in patients referred to bariatric surgery.

**Methods:**

Morbidly obese patients referred to bariatric surgery responded to the visual analogue pain scale (VAS) and the WOMAC questionnaire. X-rays of the hips and knees were evaluated. The primary endpoints were self-reported joint pain and the diagnosis of osteoarthritis by clinical and radiological criteria of the American College of Rheumatology.

**Results:**

141 patients were interviewed (85.1% women) with a mean age of 40 years. The mean body mass index was 46. The lumbar spine and knee joint were the most commonly reported as painful (77.9% and 73.2% respectively). Prevalence of knee osteoarthritis was 63.1% and hip osteoarthritis was 40.8%. Age, mean VAS and WOMAC scores were higher in the osteoarthritic individuals.

**Conclusion:**

There is prevalence of 90.1% of pain symptoms in morbidly obese patients referred to bariatric surgery. The prevalence of knee osteoarthritis was 63.1% and hip osteoarthritis was 40.8% in this sample.

## RESUMO

**Racional:**

Alto índice de massa corpórea, assim como a manutenção desta condição por longo período de tempo, são importantes fatores de risco para o desenvolvimento de osteoartrite.

**Objetivo:**

Determinar a prevalência de dor articular e osteoartrite em pacientes aguardando cirurgia bariátrica.

**Métodos:**

Pacientes obesos mórbidos responderam à escala e questionário (VAS e WOMAC) de dor e função. Radiografias dos quadris e joelhos foram avaliadas. Os desfechos primários foram dor articular referida nos questionários e o diagnóstico de osteoartrite feito através dos critérios clinicoradiológicos do Colégio Americano de Reumatologia.

**Resultados:**

Cento e quarenta e um pacientes foram entrevistados (85,1% mulheres) com idade média de 40 anos. A média do índice de massa corpórea foi de 46. Coluna lombar e joelhos foram as regiões mais comumente referidas com dor (77,9% e 73,2% respectivamente). A prevalência de osteoartrite dos joelhos foi de 63,1% e dos quadris foi de 40,8%. Idade, média da escala visual de dor e resultados do questionário de WOMAC foram maiores nos indivíduos com osteoartrite.

**Conclusão:**

Há prevalência de 90,1% de sintomas dolorosos nos pacientes obesos mórbidos encaminhados para cirurgia bariátrica. A osteoartrite dos joelhos foi de 63,1% e de quadris de 40,8%.

## INTRODUCTION

Osteoarthritis (OA), the most common form of joint disease^10^; it is a multifactorial pathology that leads to articular cartilage damage^11^ and inflammatory changes in the joint^4^ ^,^ ^21^. OA is a slow and gradual process, highly prevalent in the adult population, which causes pain, loss of function and poor quality of life, especially in the elderly and obese individuals^5^. High body mass index (BMI), as well as maintaining this condition for a long period of time, are important risk factors for its development^14^.

The World Health Organization (WHO) estimates that 10% of the population over 60 years has serious medical problems resulting from OA^20^. Lawrence et al.^10^ estimate a prevalence of 27 million people with it in the adult population of the United States. Senna et al.^16^ reported in 2004 a prevalence of 4.14% in the Brazilian population. A large increase in numbers is expected in Brazil, since Brazilian population is aging and becoming increasingly obese^5^. There is a direct relationship between the prevalence of OA and increasing age and BMI^19^. 

Obesity is also a condition that imposes heavy burden on society. Obese individuals have increased cardiovascular events and many other conditions, including osteoarthritis, which is strongly associated with high metabolic and inflammatory environments^17^. Besides the obvious mechanical overload, cytokines associated with adipose tissue, including adiponectin, leptin and resistin also influence the onset and worsening of OA through direct degradation of the joint or inflammatory processes stimulation^17^. Literature shows clear relationship between weight loss through bariatric surgery and OA symptoms improvement^1^ ^,^ ^7^ ^,^ ^8^.

Prevalence of osteoarthritis in Brazilian adult population is 4.14% according to the only existing study on the subject^16^. Obesity is a major risk factor for osteoarthritis. An American study revealed a prevalence of 22.9% of osteoarthritis in obesity surgery patients^15^. There are no national studies assessing the prevalence of joint pain and osteoarthritis in the Brazilian obese population. 

The aim of this study was to define the prevalence of joint pain and osteoarthritis of the knees and hips of obese patients awaiting treatment of obesity through bariatric surgery.

## METHODS

This is a prevalence study (cross-sectional) conducted in a bariatric surgery service between July and December 2015 and was approved by the local Research Ethics Committee.

### Patients

Were evaluated 141 consecutive patients in preoperative assessment for bariatric surgery. Inclusion criteria were obese with BMI above 35 referred for bariatric surgery and ability to read, understand and accept the informed consent.

#### *Clinical evaluation*

Patients responded to visual analogue pain scale (VAS)^3^ and Western Ontario and McMaster Universities (WOMAC) questionnaire^6^. The presence of articular pain was investigated. Anthropometric data also were collected, such as age, gender, race, height and weight, and BMI was calculated dividing weight (kg) by squared height (meters).

#### *Radiological assessment*

Patients underwent radiographs of the hips (anteroposterior incidence) and knees (unilateral weight bearing, patellar axial and lateral views). The radiographs were classified according to Kellgren and Lawrence classification^16^.

The primary outcomes were presence of self-reported joint pain and diagnosis of osteoarthritis according to the clinical and radiological American College of Rheumatology criteria for knee and hip OA^2^.

### Statistical analysis

Descriptive analysis was performed for all variables. Data was presented as mean and standard deviation (SD) for continuous variables and as absolute frequency and relative frequency for categorical variables. Kolmogorov-Smirnov test, histograms and multivariate skewness and kurtosis measures were applied to determine which variables were normally distributed. Comparisons between two continuous variables were performed using the Student-t test for independent samples and with Mann-Whitney u test when data did not meet conditions for use of parametric tests. Binary logistic regression was performed to test the association between total prevalence of OA, prevalence of knee OA and hip OA (dependent variables) and the risk factors such as age, gender, height, weight and BMI. Results were described as Odds Ratio (OR) (95% confidence intervals, p-value). Chi-square test was used for categorical variables, or Fisher’s exact test when 20% or more of the table cells showed absolute count of less than five. The level of significance was set at 0.05. All reported p values are two-tailed. All analyzes were performed using IBM SPSS Statistics software (version 22.0 Armonk, NY, IBM Corp.).

## RESULTS

### Descriptive statistics

One hundred forty-one patients were analyzed. Demographic characteristics of the sample are shown in Table 1.

**TABLE 1:** Patients demographics

	Mean?SD or Absolute frequency (relative)
Age	39.7 ? 11.5
Height	1.63 ? 0.09
Weight	123.8 ? 28.5
BMI	46.6 ? 8.9
Gender	
Male	21 (14.9%)
Female	120 (85.1%)

SD=standard deviation; BMI=body mass index

Prevalence of musculoskeletal pain in this sample of morbidly obese patients was very high. Only 14 patients (9.9%) had no complaints. The other 127 (90.1%) reported pain in one or more joints. The most common pain site was the lumbar spine, followed by the knees. Of these 127 patients with pain, 99 (77.9%) had low back pain, 33 (25.9%) dorsal pain, 33 (25.9%) cervical pain, 93 (73.2%) knee pain , 61 (48.1%) hip pain and 54 (42.5%) reported pain in the hands. Only 15 patients (11.8%) reported pain on a single joint. Moment VAS had a mean of 39.9±34.6. Last three days’s VAS was 52.4±34.1. 

**FIGURE 1:**
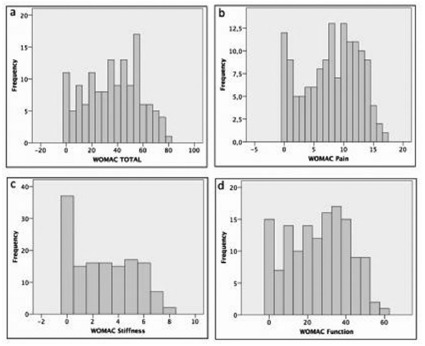
Histograms: A) WOMAC total; B) WOMAC pain subscale; C) WOMAC stiffness subscale; D) WOMAC function subscale data distribution

Mean total WOMAC was 36.3±21.2. Mean WOMAC pain subscale was 7.9±4.6. Mean WOMAC stiffness subscale was 2.8±2.4. Mean WOMAC function subscale was 25.6±15.5. The result of the total WOMAC questionnaire and its subscales (pain, stiffness and function) are shown in Figure 1.

Radiographs were taken in 103 patients. The other 38 patients refused or did not attend to the scheduled examination. Radiographs were classified according to the Kellgren-Lawrence scheme (K&L) by three authors. In case of disagreement, the classification given by the majority (two observers) was considered. There was no total disagreement.

In these 103 patients, 80 (77.7%) had one or more joints with joint pain and radiological changes consistent with OA (K&L ≥2). The prevalence of knee OA was 63.1% (n=65) and hip OA was 40.8% (n=42).

### Analytical statistics

There was no difference between the prevalence of OA and gender considering any joint (male=10, 58.8%; female=70, 81.4%; p=0.056), only knee OA (M=9, F=56; p=0.412) or only hip OA (M=4, F=38, p=0.176). Regarding the continuous variables, comparisons between the average of the group with OA (K&L ≥2) and without OA (K&L ≤1) are shown in Table 2. 

**TABLE 2:** Comparison of continuous variables between obese individuals with or without OA

	With OA (n=80)	Without OA (n=23)	p
Age	41.3 ± 12.1	35.3 ± 8.9	0.020*
Weight	121.5 ± 26.6	134.4 ± 38.2	0.193
Height	1.61 ± 0.20	1.66 ± 0.11	0.262
BMI	45.1 ± 9.3	48.1 ± 10.7	0.424
WOMAC total	41.1 ± 19.8	29.3 ± 22.9	0.017*
WOMAC pain	9.0 ± 4.3	5.7 ± 4.9	0.004*
WOMAC stiffness	3.2 ± 2.3	2.0 ± 2.2	0.026*
WOMAC function	28.9 ± 14.4	21.7 ± 16.4	0.046*
VAS moment	44.9 ± 33.6	33.7 ± 33.4	0.174
VAS 3-days	61.2 ± 30.8	38.4 ± 34.8	0.011*

*p<0.05. OA=osteoarthritis; BMI=body mass index; VAS=visual analogue scale; WOMAC=Western Ontario and Mcmaster Universities questionnaire

The age of the patients with OA was higher, as well as the WOMAC and last three days VAS levels. Associations between continuous variables as potential risk factors for developing OA tested by binary logistic regression are presented in Tables 3 and 4.

**TABLE 3:** Binary logistic regression for knee or hip OA risk factors

	OA total OR (CI); p value
Age	1.054 (1.003-1.107); p = 0.037*
Weight	1.009 (0.944-1.079); p = 0.783
Height	0.187(0.000-2.628);p = 0.731
BMI	0.972 (0.805-1.173); p = 0.766
Gender	0.302 (0.056-1.631); p = 0.166

*p<0.05. OA=osteoarthritis; OR=Odds Ratio; CI=confidence interval; BMI=body mass index

**TABLE 4:** Binary logistic regression for knee and hip OA risk factors

	Knee OA OR (CI); p value	Hip OA OR (CI); p value
Age	1.032(0.992-1.073);p=0.119	1.023(0.986-1.061);p=0.230
Weight	1.047(0.887-1.236);p=0.588	0.996(0.960-1.032);p=0.815
Height	0.000 (0.000-6.589);p=0.556	0.091(0.001-5.480);p=0.251
BMI	0.851(0.536-1.349);p=0.491	1.016(0.916-1.127);p=0.764
Gender	0.696(0.153-3.172);p=0.640	0.602(0.130-2.780);p=0.516

*p<0.05. OA=osteoarthritis; OR=Odds Ratio; CI=confidence interval; BMI=body mass index

## DISCUSSION

This study found a high prevalence of joint pain, especially in the lumbar spine and knees, as well as OA in the hips and knees in a population of morbidly obese patients awaiting for bariatric surgery. This is the first Brazilian report of prevalence of pain and OA in morbidly obese population.

Aging and obesity are the two main risk factors for OA development^5^. There is a strong tendency, therefore, of a great increase in its prevalence in our country, since according to governmental reports the Brazilian population is getting older and heavier^5^ ^,^ ^14^. The number of individuals over 60 years old increased from 7.2 million in 1980 to 19.2 million in 2010, and probably will reach 64 million in 2050^5^. Recent decades has also witnessed significant increase in population’s overweight (BMI between 25-30) and obesity (BMI above 30) rates and for the first time the number of overweight or obese people exceeded 50% of the Brazilian population^14^.

It is currently known that knee OA is strongly associated with high metabolic and inflammatory environments found in obesity^9^. Cytokines associated with adipose tissue, including adiponectin, resistin and leptin may influence OA through direct articular degradation of by enhancing local inflammatory processes. Although not all obese people will develop knee OA, it appears to be strongly associated with the coexistence of disorders of lipid and glucose metabolism. Furthermore, the higher load present on obese joints can be detected by mechanoreceptors on the surface of chondrocytes, triggering intracellular signaling cascades of cytokines, growth factors and matrix metalloproteinases^12^.

This study, as reported in the literature, found a direct relationship between age, obesity and osteoarthritis. The present analysis did not find significant statistical correlation between gender and OA, maybe be due to the low number of males in our sample. The vast majority of our subjects were female. There is increasing evidence that estrogen plays an important role in maintaining homeostasis of articular tissues. The dramatic increase in the prevalence of OA in women after menopause^18^, which is associated with the presence of estrogen receptors in articular tissues, suggests a link between OA and loss of ovarian function^18^. Although great attention is focused on estrogen’s effect on articular cartilage, its deficiency also affects other joint tissues involved in OA, like subchondral bone, synovium, muscle, ligament and capsule^13^.

Since OA is a chronic disease, prevalence measurement becomes much more important than incidence and can indicate the risk of exposure for susceptible individuals. Prevalence studies are often used for planning public health and as an initial step (baseline) to assess comorbidity control programs.

In addition to the simple prevalence of the disease, we also obtained the algofunctional profile of the studied individuals through the application of Analogue Visual Scale of pain (VAS) and WOMAC questionnaire. We could also observe greater pain and loss of function in the OA individuals, which had almost twice the pain and loss of function expressed by a higher score on the WOMAC questionnaire. It is important to notice that even individuals without OA diagnosis presented with high WOMAC scores, which points out the functional limitation imposed by the presence of obesity. Several studies have shown an improvement of symptoms and quality of life in obese osteoarthritic subjects treated with bariatric surgery^1^ ^,^ ^7^ ^,^ ^8^.

The main limitation of this study is that the sample is composed of individuals referred for surgical treatment of obesity in the public system. There could be some degree of selection bias since joint pain can act as a motivator to make the individual seek surgical treatment. Thus, the prevalence of joint pain and OA could be expected to be smaller in the general Brazilian obese population. To overcome such limitation it would be necessary to carry out a sampling study in the Brazilian population involving home visits, much more expensive and time consuming. 

Our next step will be to re-evaluate the individuals after bariatric surgery and observe any correlation between this treatment and changes in symptoms caused by osteoarthritis.

## CONCLUSION

There is a prevalence of 90.1% of pain symptoms in morbidly obese patients awaiting treatment with bariatric surgery. The prevalence of knee OA was 63.1% and hip OA was 40.8% in this sample.
